# Validity of Biomarkers of Early Circulatory Impairment to Predict Outcome: A Retrospective Analysis

**DOI:** 10.3389/fped.2019.00212

**Published:** 2019-05-29

**Authors:** María Carmen Bravo, Paloma López-Ortego, Laura Sánchez, Rosario Madero, Fernando Cabañas, Armin Koch, Héctor Rojas-Anaya, Heike Rabe, Adelina Pellicer

**Affiliations:** ^1^Department of Neonatology, La Paz University Hospital, Madrid, Spain; ^2^Division of Statistics, La Paz University Hospital, Madrid, Spain; ^3^Department of Pediatrics and Neonatology, Quironsalud Madrid University Hospital, Madrid, Spain; ^4^Medizinische Hochschule Hannover, Institut für Biometrie, Hannover, Germany; ^5^Academic Department of Paediatrics, Brighton and Sussex Medical School, Brighton, United Kingdom

**Keywords:** transitional circulation, circulatory impairment, cardiovascular treatment, hypotension, preterm

## Abstract

**Objectives:** The definition of circulatory impairment in the premature infant is controversial. Current research suggests overdiagnosis and overtreatment. We aimed to analyse which biomarkers move clinicians to initiate cardiovascular treatment (CVT). The prognostic capacity for adverse outcome (death and/or moderate-severe brain damage by cranial ultrasound at term equivalent) of these biomarkers was evaluated.

**Study Design:** Retrospective data analysis from preterm infants enrolled in a placebo-controlled trial on dobutamine for low superior vena cava (SVC) flow, who showed normal SVC flow within the first 24 h (not randomized). Five positive biomarkers were considered: MABP < gestational age (GA)-1 mmHg; MABP < GA-5 mmHg; lactate > 4 mmol/L; BE < −9 mmol/L; SVC flow <51 ml/kg/min.

**Results:** *Ninety eight* infants formed the study cohort. Thirty six received CVT (2–95 h). Logistic regression models adjusted for gestational age showed a positive association between CVT and the risk of death or moderate-severe abnormal cranial ultrasound at term equivalent [(OR 5.2, 95%CI: 1.8–15.1) *p* = 0.002]. MABP < GA-1 mmHg and lactate > 4 mmol/L were the most prevalent biomarkers at start of treatment. Low BE, high serum lactate and low SVC flow at first echocardiography showed a trend toward being associated with adverse outcome, although not statistically significant.

**Conclusions:** Low blood pressure and high lactate are the most prevalent biomarkers used for CVT prescription. Lactic acidosis and low SVC flow early after birth showed a trend toward being associated with adverse outcome. These findings support using a combination of biomarkers for inclusion in a placebo-controlled trial on CVT during transitional circulation.

## Introduction

Circulatory impairment is a common complication of the transitional circulation in the preterm infant (1). However, there is no validated scoring system to guide the management of this condition, leading to huge differences in approaches to cardiovascular treatment (CVT) among clinicians (2–5). This is a matter of the utmost importance, as several studies suggest the use of such therapies may increase the risk of adverse outcome in the most vulnerable population (6–8).

The literature increasingly supports the need of further work on the assessment of efficacy and safety of CVT routinely used in neonates; within the safeguards of high quality placebo-controlled trials (9, 10). A key question in the design of such trials is the entry criteria for randomization. Our work addresses this question firstly by investigating which biomarkers influence treatment decisions in routine clinical practice; and secondly by evaluating their diagnostic-prognostic validity for adverse outcome.

Hypotension is probably the most widely used biomarker to initiate CVT (11, 12). However, the most common pathophysiology underlying early circulatory failure during transitional circulation in the preterm infant is characterized by increased peripheral vascular resistance and increased afterload, which may cause myocardial dysfunction and impaired blood flow distribution, in spite of “normal” central blood pressure (1, 13). An alternative to using low blood pressure alone to initiate CVT is to focus on uneven blood flow distribution and poor perfusion, using a combination of clinical and biochemical parameters that indicate abnormalities of the macro- and microcirculation. The parameters selected for investigation in this work were mean arterial blood pressure (MABP), serum lactate, base excess (BE) and superior vena cava (SVC) flow measured by functional echocardiography; with the following proposed biomarker cut-off values: MABP lower than the gestational age minus 1 mmHg (MABP < GA-1 mmHg), MABP lower than the gestational age minus 5 mmHg (MABP < GA-5 mmHg), SVC flow < 51 ml/kg/min, lactate > 4 mmol/L and BE worse than −9 mmol/L (BE < −9).

Two cut-off values for MABP are being considered because there is considerable variability amongst clinicians in the threshold for intervention in the case of low blood pressure (2, 8, 20): some clinicians following a less proactive approach of permissive hypotension (6, 8), while others are not in “equipoise” and treat hypotension right away to avoid the risk of cerebral ischemia in case of impaired cerebral autoregulation. The first cut-off value represents the “best practice” approach (MABP < GA-1) which, although widely used clinically, does not constitute a proven treatment threshold. The second cut-off value (MABP < GA-5) was informed by two recent studies described next. Firstly a large German cohort study (18) that included preterm infants below 32 completed weeks, found that those whose lowest MABP during the first 24 h from birth was in the lowest quartile for gestational age (roughly, MABP < GA-5 mmHg) had an increased risk for death, intraventricular hemorrhage or bronchopulmonary dysplasia when compared with infants with minimal MABP at or above the 25th percentile. Secondly our group has recently reported a bivariate autoregressive spectral coherence method to analyse the cerebral autoregulatory capacity (19, 31). The method showed good sensitivity and specificity for identifying infants with low SVC flow, theoretically at risk of cerebral hypoperfusion, brain injury and impaired neurodevelopment (32–35). In this study, infants with impaired cerebral autoregulatory capacity were more prone to developing severe intracranial hemorrhage and death. The autoregulation classifier was associated with the percent of time of MABP < GA-5 mmHg but not with MABP < GA-1mmHg, possibly indicating the lowest range of the autoregulatory plateau. Thus the second cut-off value (MABP < GA-5) is more congruent with the pathophysiological pathway of the ischemia-reperfusion injury in our previous studies (19, 31).

The SVC flow cut-off value is derived from an internal (unpublished) reliability study for SVC flow measurement, to address the theoretical variability of the method (36, 37). The intra-observer repeatability index was 25% in our reliability study. This index is defined as the repeatability coefficient divided by the mean of all the measures (36) and indicates the percentage of change between measurements that would have a 95% probability of not occurring due to chance or measurement inaccuracy. To be sure that no infant is misdiagnosed, we favor using the cut-off value of 51 ml/k/min that represents the reported 41 ml/k/min cut-off value to define low SVC flow according to previous studies (1, 32, 34, 35) plus 25%.

The proposed cut-off values of the laboratory early biomarkers to be explored, lactate and BE, are supported by previous studies (22, 23, 29, 30). In a similar population of newborns it has been found that early hyperlactatemia (median highest lactate within first 12 h from birth was 3.86 mmol/L) associated increased risk for death (23). Both in ventilated infants (22) and in infants diagnosed with necrotizing enterocolitis (29), higher lactate early in the disease process carried increased adverse outcome.

The primary motivation of this study was to determine which of the five chosen cut-off values would qualify as biomarkers to be used as criterion for inclusion of patients in a future phase III randomized clinical trial to demonstrate efficacy and safety for dobutamine to treat hemodynamic insufficiency during transitional circulation in the preterm infant (NEO-CIRC FP7; HEALTH-2011.4.2-1) (14).

## Methods

A retrospective *ad hoc* analysis was conducted on data from a pilot randomized placebo-controlled trial of dobutamine for low superior vena cava flow (SVC flow) in preterm infants reported elsewhere (14). From a total of 127 infants born <31 weeks gestational age enrolled in the pilot trial there were 98 infants classified as the “normal flow group” after showing SVC flow ≥ 41 ml/kg/min in all scans performed during the first 24 h of life. Unlike their “low flow group” complement these 98 infants were not randomized to dobutamine or placebo and their condition was managed according to the criteria of the attending physician, but without the use of dobutamine. Data from the normal flow cohort was used to explore treatment decisions and outcomes based on the five circulatory biomarker cut-off values described in the introduction.

Prognostic capacity for combined adverse outcome of these cut-off values was evaluated at two early study time points: the immediate postnatal period of 2 h from birth, where no infant was yet on CVT; and the baseline defined as the time of the first echocardiography (Echo-D) scan within 12 h from birth, when the majority of infants had not received any CVT.

For completeness, the remainder of this section presents a brief description of assessments and intervention for both the normal and low flow groups in the pilot trial, with full details available from Bravo et al. (14).

After enrolment all infants had serial Echo-D scans at baseline, 60 min after baseline (to re-assess normal flow) and at the following times after birth: 12, 24, 48, 72, and 96 h. Heart rate, peripheral oxygen saturation (SaO_2_), blood pressure (either invasive or non-invasive), and urine output were recorded every 6 h during the first 96 h from birth. Biochemical parameters (pH, blood gases, and serum lactate) were obtained when clinically indicated. Infants with SVC flow < 41 ml/kg/min at any time during the first 24 h of life (low flow group) were randomized to receive dobutamine or placebo; whilst infants with SVC flow ≥ 41 ml/kg/min in all the scans performed during the first 24 h of life (normal flow group) were not randomized but continued in the trial for further assessment and follow-up and are the subject of this report. The low flow group was excluded from this retrospective analysis because it had a systematic and differential intervention based on Echo-D findings that could eventually influence their outcomes.

For all the infants (normal flow or low flow) open-label treatment with other cardiovascular support that did not include dobutamine was allowed if indicated by the attending physician and was guided by a constellation of routine clinical or biochemical data, following a standardized treatment protocol: dopamine (first line), epinephrine (second line), plus/minus volume expansion or hydrocortisone (15–17).

Main neonatal clinical outcomes were recorded at term equivalent, together with combined adverse outcome defined as: death; or moderate-severe abnormal cranial ultrasound according to term equivalent ultrasound findings (intraventricular hemorrhage grade 3; or periventricular hemorrhagic infarct; or white matter damage, defined as persistent periventricular echogenicity with/without associated ventriculomegaly or cystic periventricular leukomalacia) (15, 17). The study was approved by the hospital's Ethics Committee.

### Statistics

Quantitative (numerical) data are described as mean (SD), median (IQR), and qualitative (categorical) data as counts and percentages.

The Mann-Whitney rank-sum test and the Fisher's exact test were used for quantitative and qualitative data analysis, respectively. The CVT effect and threshold biomarkers on binary outcomes were assessed using Binary Logistics Regression Analysis (unadjusted and adjusted for GA). The odds ratio and the 95% confidence interval are reported. The CVT effect on the quantitative outcome variables was assessed using an analysis of covariance (ANCOVA) using the CVT as factor and GA age as covariate. SAS 9.3 (SAS Institute, Cary, NC USA) was used for analyses.

## Results

### Patients

The median postnatal age at the first Echo-D (definition of baseline) of the 98 patients in the normal flow group was 4 (3) h. Among them, 36 infants were judged to suffer circulatory impairment by the attending physician and received open-label CVT (volume expansion or catecholamine or both, plus/minus hydrocortisone) at a median postnatal age of 12.2 (5.4–23.2) h. For those receiving only catecholamines (*n* = 17), the median postnatal age at start on treatment was 12.5 (11–24) h. Infants who received CVT were less mature, and had lower birth weight and poorer condition at birth (Table 1). Outcomes at term equivalent were also worse in these infants (Table 2) compared to those who did not receive CVT. Logistic regression models adjusted for gestational age showed that CVT (any type) and CVT (only catecholamines) were associated with an increased risk of combined adverse outcome (death or moderate-severe abnormal cranial ultrasound according to term equivalent ultrasound findings) [(OR 5.2, 95%CI: 1.8–15.1) *p* = 0.002] and [(OR 9.7, 95%CI: 2.6–36.5) *p* = 0.001], respectively. Severe ischemic event (defined as spontaneous intestinal perforation or vascular spasm) and posthemorrhagic hydrocephalus were observed in 2 and 3 infants, respectively, who received CVT. However, the logistic regression analysis adjusted for gestational age could not be done for them as no infants without CVT showed these outcomes.

**Table 1:** Perinatal condition of the study population.

	**CVT (any type) *n* = 36**	**CVT (only Cat) *n* = 17**	**No CVT *n* = 62**	***p-*****value**	
				**CVT (any type) vs. no CVT**	**CVT (only Cat) vs. no CVT**
GA, weeks (mean, SD)	26.4 (1.7)	27.0 (1.4)	28.5 (1.8)	0.000	0.002
BW, g (mean, SD)	876 (284)	835 (284)	1,105 (312)	0.000	0.002
Maternal age, years (mean, SD)	32.6 (5.6)	32.6 (5.2)	32.7 (6.4)	0.821	0.971
Apgar 5 min < 5, *n* (%)	4 (11.1)	4 (23.5)	2 (3.5)	0.188	0.018
SNAPPE-II > 45, *n* (%)	16 (45.7)	11 (68.8)	5 (8.2)	0.000	0.000
Cord pH, *n* (%)	7.22 (0.2)	7.17 (0.2)	7.29 (0.1)	0.368	0.025
PROM > 24 h, *n* (%)	9 (25)	2 (11.8)	13 (21)	0.802	0.503
Advanced resuscitation, *n* (%)^*^	22 (61.1)	12 (70.6)	11 (17.7)	0.000	0.000
Chorioamnionitis, *n* (%)^†^	9 (25)	3 (17.6)	12 (19.4)	0.611	1
Antenatal steroids, *n* (%)*^z^*	25 (69.4)	11 (64.7)	56 (90.3)	0.012	0.018
Maternal hypertension, *n* (%)	5 (13.9)	4 (23.5)	10 (16.1)	1	0.486
SGA, *n* (%)	7 (19.4)	6 (35.3)	12 (19.4)	1	0.197
Multiple, *n* (%)	5 (13.9)	3 (17.6)	20 (32.3)	0.056	0.367
Male, *n* (%)	26 (72.2)	12 (70.6)	29 (46.8)	0.020	0.104
C-section, *n* (%)	24 (66.7)	15 (88.2)	44 (71)	0.657	0.212

*Cardiovascular treatment (CVT), volume expansion and/or catecholamine (dopamine, epinephrine) plus/minus hydrocortisone; Cat, catecholamine; SGA, small for gestational age; SNAPPE-II, Score for Neonatal Acute Physiology Perinatal Extension-II (38), PROM, premature rupture of membranes*.

* *Intubation in the delivery room with or without assisted circulation*.

† *Histological chorioamnionitis*.

Z *Completed course*.

**Table 2:** Main neonatal clinical outcomes at term equivalence.

	**CVT (any type) *n* = 36**	**CVT (only Cat) *n* = 17**	**No CVT *n* = 62**	***p-*****value**	
				**CVT (any type) vs. no CVT**	**CVT (only Cat) vs. no CVT**
Mortality, *n* (%)	7 (19.4)	2 (11.8)	2 (3.2)	0.3	0.4
Combined adverse outcome, *n* (%)	26 (72.2)	13 (76.4)	21 (33.9)	0.002	0.001
Normal cUS, *n* (%)	10 (27.8)	4 (23.5)	42 (67.7)	0.02	0.01
IVH grade 3 or 4, *n* (%)	10 (27.8)	6 (35.3)	1 (1.6)	0.01	0.005
White matter damage, *n* (%)	12 (33.3)	7 (41.2)	7 (11.3)	0.09	0.04
NEC, *n* (%)	5 (13.9)	1 (5.9)	8 (12.9)	0.8	0.4
Age at full enteral feeds in days (mean, SD)	24.5 (13)	26.5 (14)	16 (14)	0.2	0.1
PDA treated, *n* (%)	24 (66.7)	14 (82.4)	17 (27.4)	0.3	0.2
Mechanical ventilation in days (mean, SD)	44 (38)	52 (36)	8.9 (13)	0.000	0.000
O2 dependency at 36 weeks, *n* (%)	18 (50)	11 (64.7)	5 (8.1)	0.001	0.000
Laser therapy for ROP, *n* (%)	9 (25)	4 (23.5)	3 (4.8)	0.1	0.09
Age at discharge in days (mean, SD)	109.6 (48)	118 (55)	61.6 (24)	0.000	0.000

*Logistic regression analysis adjusted by gestational age*.

*Cardiovascular treatment (CVT), volume expansion and/or catecholamine (dopamine, epinephrine); Cat, catecholamine; combined adverse outcome was defined as death or moderate-severe brain damage according to the ultrasound exam performed at term equivalent [intraventricular hemorrhage grade 3; or periventricular hemorrhagic infarct; or white matter damage (persistent periventricular echogenicity with/without ventriculomegaly or cystic periventricular leukomalacia)]; IVH, intraventricular hemorrhage; ROP, retinopathy of prematurity*.

### Biomarkers and Cardiovascular Treatment Prescription (Figure 1)

The extent to which having a positive biomarker (according to the predefined cut-off values) influenced treatment decision is shown in Table 3. High serum lactate > 4 mmol/L and hypotension (MABP < GA-1) were the most prevalent biomarkers at the time CVT was started. Having at least one positive biomarker throughout the first 96 h of birth was more common among those infants who received CVT (75%) than in the non-treated infants (21%) (*p* < 0.01).

**Figure 1:**
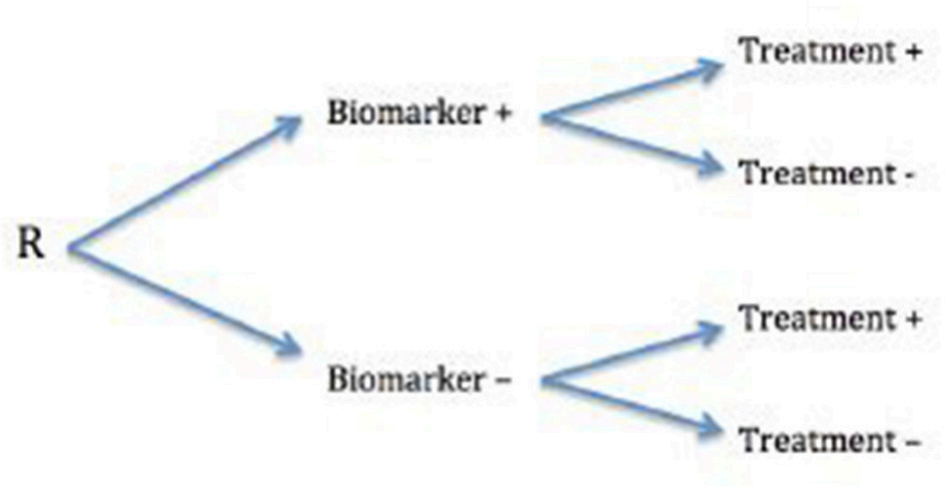
Retrospective assessment (R) of biomarkers influencing treatment decisions.

**Table 3:** Biomarker status at start of cardiovascular treatment.

**Biomarker *n* (%)**	**CVT** **(any type)** ***n* = 36**	**CVT** **(only Cat)** ***n* = 17**	**No CVT** ***n* = 62**	***p-*****value**	
				**CVT** **(any type)** **vs.** **no CVT**	**CVT** ** (only Cat)** ** vs.** ** no CVT**
MABP < GA-1 mmHg	10 (27.7)	8 (47)	7 (11.3)	0.049	0.002
MABP < GA-5 mmHg	2 (5.5)	2 (11.8)	1 (1.6)	0.55	0.11
Lactate > 4 mmol/L	11 (30.6)	8 (47)	2 (3.2)	0.000	0.000
BE < −9 mmol/L	4 (11.1)	4 (23.5)	3 (4.8)	0.42	0.03

*Cardiovascular treatment (CVT), volume expansion and/or catecholamine (dopamine, epinephrine plus/minus hydrocortisone); Cat, catecholamine*.

### Biomarkers and Outcome at Term Equivalent

High serum lactate levels at any time during the first 96 h from birth were associated with an increased risk of death (*p* = 0.002) and combined adverse outcome (*p* = 0.02). In addition, BE < −9 mmol/L was associated with increased risk of death (*p* = 0.01).

Table 4 shows results of the prognostic value for combined adverse outcome of the explored biomarkers at both the immediate postnatal and the baseline; with the combined adverse outcome at term equivalent serving as the dependent variable and the birth/baseline measurements for each biomarker serving as independent variables. The following biomarkers measured at baseline showed trends toward an increased risk of adverse outcome, but with no association with adverse outcome since all 95% CI span the null value (OR = 1) and *p* values were not statistically significant: Base excess < −9, serum lactate > 4 mmol/L, and SVC flow < 51 ml/kg/min. Results were the same after adjusting by gestational age.

**Table 4:** Association between early positive biomarker and combined adverse outcome in the study population.

		**Biomarkers at birth (first 2h of life)**				Biomarkers at baseline^*^ **(time of first Echo-D)**			
**Biomarkers (BM)**		**Adverse outcome*****^§^***		**OR (95%CI)**	***p-*value**	**Adverse outcome*****^§^***		**OR (95%CI)**	***p-*value**
		**Yes**	**No**			**Yes**	**No**		
SVC flow < 51 (*n* = 0 at birth, *n* = 98 at baseline)	BM+	–	–	–	–	5/8 (62.5)	3/8 (37.5)	4.1 (0.9–18.4)	0.10
	BM–	–	–			26/90 (28.9)	64/90 (71.1)		
MABP < GA-1 (*n* = 89 at birth, *n* = 93 at baseline)	BM+	6/26 (23.1)	20/26 (76.9)	0.6 (0.21–1.7)	0.45	7/17 (41.2)	10/17 (58.8)	1.9 (0.6–5.8)	0.2
	BM–	21/63 (33.3)	42/63 (66.7)			20/76 (26.3)	56/76 (73.7)		
MABP < GA-5 (*n* = 89 at birth, *n* = 93 at baseline)	BM+	2/8 (25)	6/8 (75)	0.7 (0.14–3.9)	1.00	2/2 (100)	(0) 0/2	Z	0.08
	BM–	25/81 (30.9)	56/81 (69.1)			25/91 (27.5)	66/91 (72.5)		
Lactate > 4 mmol/L (*n* = 90 at birth, *n* = 93 at baseline)	BM+	11/25 (44)	14/25 (56)	2.2 (0.85–5.8)	0.13	9/18 (50)	9/18 (50)	2.6 (0.9–7.4)	0.09
	BM–	17/65 (26.2)	48/65 (73.8)			21/75 (28)	54/75 (72)		
BE < −9 mmol/L (*n* = 88 at birth, *n* = 97 at baseline)	BM+	5/11 (45.5)	6/11 (54.5)	2.08 (0.58–7.5)	0.30	5/9 (55.6)	4/9 (44.4)	3.15 (0.8–12.7)	0.13
	BM–	22/77 (28.6)	55/77 (71.4)			25/88 (28.4)	63/88 (71.6)		

§ *Adverse Outcome is defined as death or intraventricular hemorrhage grade 3 or periventricular hemorrhagic infarction or white matter damage*.

* *Baseline, time at first Echo-D [5 (3) h from birth]. Study biomarkers are not always documented in all the study time points*.

*^Z^OR could not be calculated as all the infants with MABP < GA-5 presented adverse outcome*.

## Discussion

This retrospective pilot study has found that low blood pressure and high serum lactate levels were the biomarkers more frequently present at the start of CVT. Of note, BE worse than −9, serum lactate > 4 mmol/L, and SVC flow < 51 ml/kg/min, showed a trend toward increased likelihood of combined adverse outcome in this series, although this did not reach statistical significance.

The use of a variety of clinical signs, mainly management of blood pressure, to initiate CVT in the hope of maintaining adequate organ blood flow, has possibly led to overdiagnosis and overtreatment in the preterm infants. This is extremely important as the prescribed medicines could cause more harm than benefit (6–8, 20). Or it may be the case that, the disease causing the circulatory disturbance, the resultant abnormalities in blood flow distribution, or both, irrespective of treatment, could be the main determinants of the increased risk for adverse outcome in these infants (21).

There is no doubt that studies to define which infants would benefit from treatment or not are of utmost relevance (5). Hypotension, whatever the definition used, is the most frequent clinical parameter used to initiate treatment (3, 4, 11, 22); as also shown in the present study. However, a recent study revealed that irrespective of early changes in blood pressure, extremely preterm infants who received antihypotensive therapy in the first 24 h after birth had a significantly higher rate of death or impaired neurodevelopment at 2 years than those who were not treated, after controlling for illness severity (8).

The second important reflection is about the CVT to be chosen in a given condition. The distribution of cardiac output is dependent on the interplay among the various components of the cardiovascular system, both cardiac and extracardiac factors. The cardiovascular drugs, particularly catecholamines, act at different parts of this complex system, exerting different pharmacodynamic effects (10, 11). So far, even if we are able to identify who would benefit from treatment, if the pharmacodynamic effect of the chosen drug does not fit the pathophysiology of the condition, it could be counterproductive, impairing blood flow distribution or oxygen transport.

Thus, our proposal to use a combination of biomarkers addresses the possible failure of both the macro- and microcirculation. Such an approach has been used in previous observational studies (6). The macrocirculation is evaluated at the vascular (blood pressure) and at the heart level (SVC flow as an estimate of cardiac output). The adequacy of the microcirculation is proposed to be assessed by the use of lactate and BE as surrogates of tissue oxygen delivery and extraction (22, 23). It is true that sites differ in facilities and protocols to implement this in a standardized way, but considering using a combination of these biomarkers would probably help them better define both the clinical picture and the condition to address. We support the use of these “objective” biomarkers instead of other routine clinical parameters such as tachycardia, urine output, capillary refill time, or core-to-peripheral temperature gap; which seem to have a higher variability, are more influenced by external factors and have non-existent or too wide normative values for the target population (24–28).

Although negative BE has been an insensitive indicator of raised lactate concentrations in other papers (22), we observed that infants with combined adverse outcome presented more frequently a trend toward a negative BE worse than −9 mmol/L although not statistically significant. Cotside measurement of serum lactate is currently feasible in most NICUs, so this biomarker can be proposed as a routine biochemical tool.

As previously reported (8, 19) we have found an increased risk of death if any CVT is used, the odds being higher if catecholamines are added to the treatment. However, this just shows an association and not a cause-and-effect. So far, no randomized clinical trial to reach definitive conclusions has been published.

This work has explored the role of the proposed cut-off values as prognostic biomarkers. One limitation is that, because CVT in the normal flow group did not follow a specific protocol, it is difficult to elucidate if adverse outcome is caused by processes related to the biomarkers or as an effect of treatment. Data from a randomized controlled trial with dobutamine will help confirm their predictive value for treatment with this drug, or if they can be validated in other capacity for clinical trial purposes, e.g., safety, monitoring, pharmacodynamic/response, or other type of biomarker. Note that it may very well be the case that these biomarkers are prognostic (i.e., they indicate poor prognosis) but not predictive (i.e., they do not identify patients that may benefit from treatment). Another limitation of this study is the fact that a more in depth analysis to explore whether potential confounders (SNAPPE scores, advanced resuscitation, cord pH, antenatal steroids…) could have had an impact on the observed association between CVT and adverse outcome, was not done. However, CVT (any type) and CVT (only catecholamines) showed a positive association with the risk of death or moderate-severe abnormal cranial ultrasound at term equivalent adjusted for gestational age. Thus, it was considered that the most powerful confounding factor was taken in consideration and this is of great interest in itself.

For this work we used the BEST (Biomarkers, EndpointS, and other Tools) resource from the FDA-NIH Biomarker Working Group, aiming to be as consistent as possible in the use of definitions and key terms around biomarkers. We believe this is needed for true effective communication, evaluation and interpretation of scientific evidence in this field of research (39).

## Conclusions

This retrospective pilot analysis has found that high serum lactate levels and low blood pressure are the most prevalent biomarkers used to prescribe CVT in this cohort of preterm infants. In addition to low SVC flow, high lactate serum levels and worse BE showed a trend toward being associated with adverse outcome. The findings support the use of these prognostic biomarkers as entry criterion in randomized placebo-controlled trials on cardiovascular support for circulatory impairment during the transitional circulation in the preterm infant.

## Ethics Statement

The Ethics Committee of La Paz University Hospital approved the study.

## Author Contributions

MB conceptualized and designed the study, drafted the initial manuscript, collected the information from the medical charts, performed the initial analyses and approved the final manuscript as submitted. PL-O and LS collected the information from the medical charts and approved the final manuscript as submitted. RM performed the statistical analysis, reviewed the manuscript and approved the final manuscript as submitted. FC and AP conceptualized and designed the study, drafted the initial manuscript and approved the final manuscript as submitted. AK participated in the design of the statistical analysis, reviewed the manuscript and approved the final manuscript as submitted. HR-A and HR conceptualized the manuscript and approved the final manuscript as submitted.

*Neo-Circulation Expert Advisory Board:* Tonse N. K. Raju, *Bethesda, USA*; Nicholas Evans, *Sydney, Australia*; Stephanie Läer, *Düsseldorf, Germany*; Silke Mader, *Karlsfeld, Germany*; Monika Seibert-Grafe, *Mainz, Germany; Adrian Toma, Bucharest, Romania*.

*Neo-Circulation Data Monitoring Committee:Gorm Greisen,Copenhagen, Denmark; Lena Hellström-Westas,Uppsala, Sweden; Josef Högel,Ulm, Germany]*.

We also acknowledge the SAMID network (RETICS funded by the PN 2018-2011 (Spain), ISCIII-Sub-Directorate General for Research Assessment and Promotion and European Regional Development Fund (FEDER), ref. RD12/0026 and the Spanish Ministry of Health (SAS/2481/2009) for the scientific and financial support, respectively.

### Conflict of Interest Statement

The authors declare that the research was conducted in the absence of any commercial or financial relationships that could be construed as a potential conflict of interest.

## Abbreviations

BE: base excess
CVT: cardiovascular treatment
GA: gestational age
MABP: mean arterial blood pressure
SVC: superior vena cava.
